# Evolution and Emerging Trends in Depression Research From 2004 to 2019: A Literature Visualization Analysis

**DOI:** 10.3389/fpsyt.2021.705749

**Published:** 2021-10-29

**Authors:** Hui Wang, Xuemei Tian, Xianrui Wang, Yun Wang

**Affiliations:** ^1^School of Traditional Chinese Medicine, Beijing University of Chinese Medicine, Beijing, China; ^2^School of Life Sciences, Beijing University of Chinese Medicine, Beijing, China

**Keywords:** depression, major depressive disorder, bibliometrics, visual analysis, knowledge graphs, CiteSpace

## Abstract

Depression has become a major threat to human health, and researchers around the world are actively engaged in research on depression. In order to promote closer research, the study of the global depression knowledge map is significant. This study aims to map the knowledge map of depression research and show the current research distribution, hotspots, frontiers, and trends in the field of depression research, providing researchers with worthwhile information and ideas. Based on the Web of Science core collection of depression research from 2004 to 2019, this study systematically analyzed the country, journal, category, author, institution, cited article, and keyword aspects using bibliometric and data visualization methods. A relationship network of depression research was established, highlighting the highly influential countries, journals, categories, authors, institutions, cited articles, and keywords in this research field. The study identifies great research potential in the field of depression, provides scientific guidance for researchers to find potential collaborations through collaboration networks and coexistence networks, and systematically and accurately presents the hotspots, frontiers, and shortcomings of depression research through the knowledge map of global research on depression with the help of information analysis and fusion methods, which provides valuable information for researchers and institutions to determine meaningful research directions.

## Introduction

Health issues are becoming more and more important to people due to the continuous development of health care. The social pressures on people are becoming more and more pronounced in a social environment that is developing at an increasing rate. Prolonged exposure to stress can have a negative impact on brain development (1), and depression is one of the more typical disorders that accompany it. Stress will increase the incidence of depression (2), depression has become a common disease (3), endangering people's physical health. Depression is a debilitating mental illness with mood disorders, also known as major depression, clinical depression, or melancholia. In human studies of the disease, it has been found that depression accounts for a large proportion of the affected population. According to the latest data from the World Health Organization (WHO) statistics in 2019, there are more than 350 million people with depression worldwide, with an increase of about 18% in the last decade and an estimated lifetime prevalence of 15% (4), it is a major cause of global disability and disease burden (5), and depression has quietly become a disease that threatens hundreds of millions of people worldwide.

Along with the rise of science communication research, the quantification of science is also flourishing. As a combination of “data science” and modern science, bibliometrics takes advantage of the explosive growth of research output in the era of big data, and uses topics, authors, publications, keywords, references, citations, etc. as research targets to reveal the current status and impact of the discipline more accurately and scientifically. Whereas, there is not a wealth of bibliometric studies related to depression. Fusar-Poli et al. (6) used bibliometrics to systematically evaluate cross-diagnostic psychiatry. Hammarström et al. (7) used bibliometrics to analyze the scientific quality of gender-related explanatory models of depression in the medical database PubMed. Tran et al. (8) used the bibliometric analysis of research progress and effective interventions for depression in AIDS patients. Wang et al. (9) used bibliometric methods to analyze scientific studies on the comorbidity of pain and depression. Shi et al. (10) performed a bibliometric analysis of the top 100 cited articles on biomarkers in the field of depression. Dongping et al. (11) used bibliometric analysis of studies on the association between depression and gut flora. An Chunping et al. (12) analyzed the literature on acupuncture for depression included in PubMed based on bibliometrics. Yi and Xiaoli (13) used a bibliometric method to analyze the characteristics of the literature on the treatment of depression by Chinese medicine in the last 10 years. Zhou and Yan (14) used bibliometric method to analyze the distribution of scientific and technological achievements on depression in Peoples R China. Guaijuan (15) performed a bibliometric analysis of the interrelationship between psoriasis and depression. Econometric analysis of the relationship between vitamin D deficiency and depression was performed by Yunzhi et al. (16) and Shauni et al. (17) performed a bibliometric analysis of domestic and international research papers on depression-related genes from 2003 to 2007. A previous review of depression-related bibliometric studies revealed that there is no bibliometric analysis of global studies in the field of depression, including country network analysis, journal network analysis, category network analysis, author network analysis, institutional network analysis, literature co-citation analysis, keyword co-presentation analysis, and cluster analysis.

The aim of this study was to conduct a comprehensive and systematic literature-based data mining and metrics analysis of depression-related research. More specifically, this analysis focuses on cooperative network and co-presentation analysis, based on the 36,477 papers included in the Web of Science Core Collection database from 2004 to 2019, and provides an in-depth analysis of cooperative network, co-presentation network, and co-citation through modern metrics and data visualization methods. Through the mining of key data, the data correlation is further explored, and the results obtained can be used to scientifically and reasonably predict the depression-related information. This study aims to show the spatial and temporal distribution of research countries, journals, authors, and institutions in the field of depression in a more concise manner through a relational network. A deeper understanding of the internal structure of the research community will help researchers and institutions to establish more accurate and effective global collaborations, in line with the trend of human destiny and globalization. In addition, the study will allow for the timely identification of gaps in current research. A more targeted research direction will be established, a more complete picture of the new developments in the field of depression today will be obtained, and the research protocol will be informed for further adjustments. The results of these analyses will help researchers understand the evolution of this field of study. Overall, this paper uses literature data analysis to find research hotspots in the field of depression, analyze the knowledge structure within different studies, and provide a basis for predicting research frontiers. This study analyzed the literature in the field of depression using CiteSpace 5.8.R2 (64-bit) to analyze collaborative networks, including country network analysis, journal network analysis, category network analysis, researcher network analysis, and institutional network analysis using CiteSpace 5.8.R2 (64-bit). In addition, literature co-citation, keyword co-presentation, and cluster analysis of depression research hotspots were also performed. Thus, exploring the knowledge dimensions of the field, quantifying the research patterns in the field, and uncovering emerging trends in the field will help to obtain more accurate and complete information. The large amount of current research results related to depression will be presented more intuitively and accurately with the medium of information technology, and the scientific evaluation of research themes and trend prediction will be provided from a new perspective.

## Methods

### Data Sources

The data in this paper comes from the Web of Science (WoS) core collection. The time years were selected as 2004–2019. First, the literature was retrieved after entering “depression” using the title search method. A total of 73,829 articles, excluding “depression” as “suppression,” “decline,” “sunken,” “pothole,” “slump,” “low pressure,” “frustration.” The total number of articles with other meanings such as “depression” was 5,606, and the total number of valid articles related to depression was 68,223. Next, the title search method was used to search for studies related to “major depressive disorder” not “depression,” and a total of 8,070 articles were retrieved. For the two search strategies, a total of 76,293 records were collected. The relevant literature retrieved under the two methods were combined and exported in “plain text” file format. The exported records included: “full records and references cited.” CiteSpace processed the data to obtain 41,408 valid records, covering all depression-related research articles for the period 2004–2019, and used this as the basis for analysis.

### Processing Tools

CiteSpace (18), developed by Chao-Mei Chen, a professor in the School of Information Science and Technology at Drexel University, is a Java-based program with powerful data visualization capabilities and is one of the most widely used knowledge mapping tools. The software version used in this study is CiteSpace 5.8.R2 (64-bit).

### Methods of Analysis

This study uses bibliometrics and data visualization as analytical methods. First, the application of bibliometrics to the field of depression helped to identify established and emerging research clusters, demonstrating the value of research in this area. Second, data visualization provides multiple perspectives on the data, presenting correlations in a clearer “knowledge graph” that can reveal underestimated and overlooked trends, patterns, and differences (19). CiteSpace is mainly based on the “co-occurrence clustering idea,” which extracts the information units (keywords, authors, institutions, countries, journals, etc.) in the data by classification, and then further reconstructs the data in the information units to form networks based on different types and strengths of connections (e.g., keyword co-occurrence, author collaboration, etc.). The resulting networks include nodes and links, where the nodes represent the information units of the literature and the links represent the existence of connections (co-occurrence) between the nodes. Finally, the network is measured, statistically analyzed, and presented in a visual way. The analysis needs to focus on: the overall structure of the network, key nodes and paths. The key evaluation indicators in this study are: betweenness centrality, year, keyword frequency, and burst strength. Betweenness centrality (BC) is the number of times a node acts as the shortest bridge between two other nodes. The higher the number of times a node acts as an “intermediary,” the greater its betweenness centrality. Betweenness centrality is a measure of the importance of articles found and measured by nodes in the network by labeling the category (or authors, journals, institutions, etc.) with purple circles. There may be many shortest paths between two nodes in the network, and by counting all the shortest paths of any two nodes in the network, if many of the shortest paths pass through a node, then the node is considered to have high betweenness centrality. In CiteSpace, nodes with betweenness centrality over 0.1 are called critical nodes. Year, which represents the publication time of the article. Frequency, which represents the number of occurrences. Burst strength, an indicator used to measure articles with sudden rise or sudden decline in citations. Nodes with high burst strength usually represent a shift in a certain research area and need to be focused on, and the burst article points are indicated in red. The nodes and their sizes and colors are first analyzed initially, and further analyzed by betweenness centrality indicators for evaluation. Each node represents an article, and the larger the node, the greater the frequency of the keyword word and the greater the relevance to the topic. Similarly, the color of the node represents time: the warmer the color, the more recent the time; the colder the color, the older the era; the node with a purple outer ring is a node with high betweenness centrality; the color of each annual ring can determine the time distribution: the color of the annual ring represents the corresponding time, and the thickness of one annual ring is proportional to the number of articles within the corresponding time division; the dominant color can reflect the relative concentration of the emergence time; the node The appearance of red annual rings in the annual rings means hot spots, and the frequency of citations has been or is still increasing rapidly.

## Large-Scale Assessment

### Country Analysis

**Figure 1:**
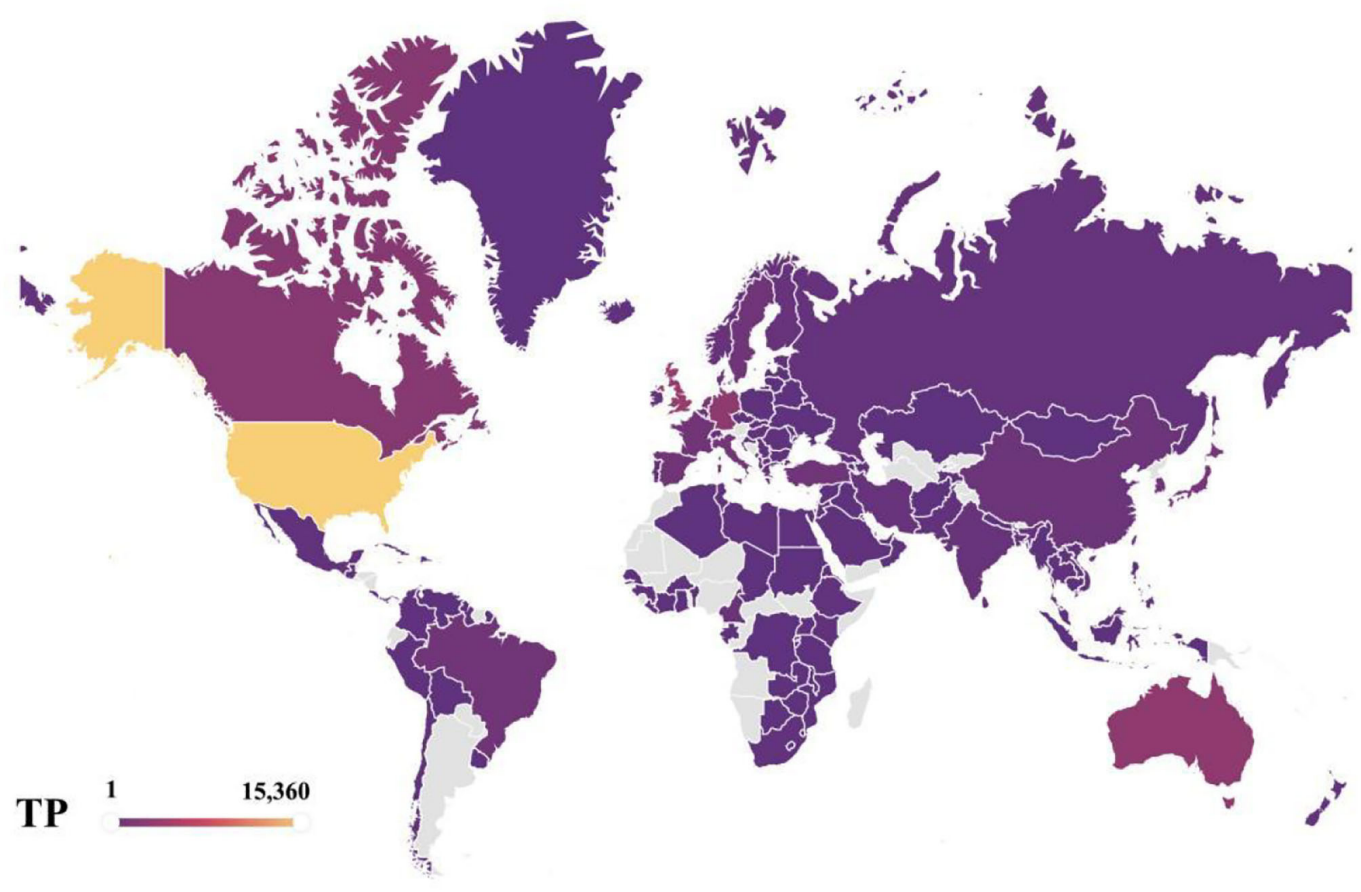
Geographical distributions of publications, 2004–2019.

During the period 2004–2019, a total of 157 countries/territories have conducted research on depression, which is about 67.38% of 233 countries/territories worldwide. This shows that depression is receiving attention from many countries/regions around the world. Figure 1 shows the geographical distribution of published articles for 157 countries. The top 15 countries are ranked according to the number of articles published. Table 1 lists the top 15 countries with the highest number of publications in the field of depression worldwide from 2004 to 2019. These 15 countries include 4 Asian countries (Peoples R China, Japan, South Korea, Turkey), 2 North American countries (USA, Canada), 1 South American country (Brazil), 7 European countries (UK, Germany, Netherlands, Italy, France, Spain, Sweden), and 1 Oceania country (Australia).

**Table 1:** The top 15 productive countries.

**Rank**	**Countries**	**TP**	**TP R (%)**	**TPA (million)**	**TPA R (%)**	**BC**	**Year**
1	USA	15,360	27.13	7.87	0.20	0.98	2004
2	UK	3,852	6.80	1.84	0.21	0.20	2004
3	Peoples R China	3,802	6.72	4.63	0.08	0.01	2005
4	Australia	3,243	5.73	0.93	0.35	0.02	2004
5	Germany	3,156	5.57	1.89	0.17	0.09	2006
6	Canada	2,679	4.73	1.17	0.23	0.09	2004
7	Netherlands	2,146	3.79	0.67	0.32	0.03	2006
8	Japan	1,553	2.74	1.54	0.10	0.05	2007
9	Italy	1,447	2.56	1.18	0.12	0.08	2007
10	South Korea	1,304	2.30	0.84	0.16	0.03	2007
11	France	1,223	2.16	1.29	0.09	0.10	2007
12	Spain	1,165	2.06	0.95	0.12	0.01	2007
13	Brazil	1,154	2.04	0.65	0.18	0.07	2007
14	Turkey	1,112	1.96	0.45	0.25	0.00	2007
15	Sweden	1,066	1.88	0.44	0.24	0.01	2005

*TP, total publications; TP R (%), the ratio of the amount of the publications in the country to the publications in the word during 2004–2019; BC, betweenness centrality; TPA (million), total publications in all areas; TPA R (%), the ratio of the amount of publications in depression to publications in all areas*.

Overall, the main distribution of these articles is in USA and some European countries, such as UK, Germany, Netherlands, Italy, France, Spain, and Sweden. This means that these countries are more interested and focused on research on depression compared to others. The total number of publications across all research areas in the Web of Science core collection is similar to the distribution of depression research areas, with the trend toward USA, UK, and Peoples R China as leading countries being unmistakable, and USA has been a leader in the field of depression, with far more articles published than any other country. It can also be seen that USA is the country with the highest betweenness centrality in the network of national collaborations analyzed in this paper. USA research in the field of depression is closely linked to global research, and is an important part of the global collaborative network for depression research. As of 2019, the total number of articles published in depression performance research in USA represents 27.13% of the total number of articles published in depression worldwide, which is ~4 times more than the second-place country, UK, which is far ahead of other countries. Peoples R China, as the third most published country, has a dominant number of articles, but its betweenness centrality is 0.01, reflecting the fact that Peoples R China has less collaborative research with other countries, so Peoples R China should strengthen its foreign collaborative research and actively establish global scientific research partnerships to seek development and generate breakthroughs in cooperation. The average percentage of scientific research on depression in each country is about 0.19%, also highlighting the urgent need to address depression as one of the global human health problems. The four Asian countries included in the top 15 countries are Peoples R China, Japan, South Korea, and Turkey, with Peoples R China ranking third with 6.72% of the total number of all articles counted. The distribution may be explained by the fact that Peoples R China is the largest developing country with a rapid development rate as the largest. Along with the steady rise in the country's economic power, people are creating economic benefits and their health is becoming a consumable commodity. The lifetime prevalence and duration of depression varies by country and region (2), but the high prevalence and persistence of depression worldwide confirms the increasing severity of the disease worldwide. The WHO estimates that more than 300 million people, or 4.4% of the world's population, suffer from depression (20), with the number of people suffering from depression increasing at a patient rate of 18.4% between 2005 and 2015. Depression, one of the most prevalent mental illnesses of our time, has caused both physical and psychological harm to many people, and it has become the leading cause of disability worldwide today, and in this context, there is increased interest and focus on research into depression. It is expected that a more comprehensive understanding of depression and finding ways to prevent and cope with the occurrence of this disease can help people get rid of the pain and shadow brought by depression, obtain a healthy and comfortable physical and mental environment and physical health, and make Chinese contributions to the cause of human health. Undoubtedly, the occurrence of depressive illnesses in the context of irreversible human social development has stimulated a vigorous scientific research environment on depression in Peoples R China and other developing countries and contributed to the improvement of research capacity in these countries. Moreover, from a different perspective, the geographical distribution of articles in this field also represents the fundamental position of the country in the overall scientific and academic research field.

### Growth Trend Analysis

**Figure 2:**
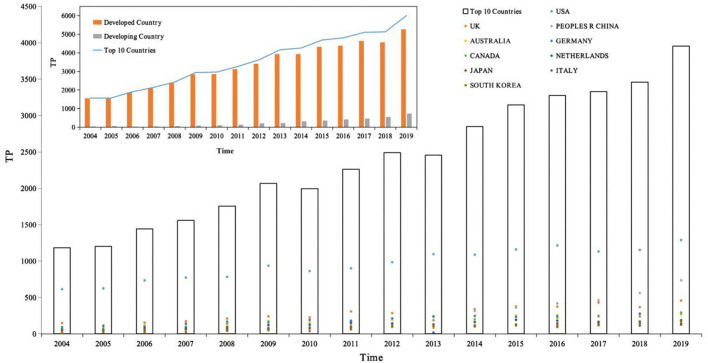
The distribution of publications in top 10 productive countries, 2004–2019. Source: author's calculation. National development classification criteria refer to “Human Development Report 2020” (21).

Figure 2 depicts the distribution of 38,433 articles from the top 10 countries in terms of the number of publications and the trend of growth during 2004–2019.

First, the number of articles published per year for the top 10 countries in terms of productivity was counted and then the white bar chart in Figure 2 was plotted, with the year as the horizontal coordinate and total publications as the vertical coordinate, showing the distribution of the productivity of articles in the field of depression per year. The total number of publications for the period 2004–2019 is 38,433. Based on the white bars and line graphs in Figure 2, we can divide this time period into three growth periods. The number of publications in each growth period is calculated based on the number of publications per year. As can be seen from the figure, the period 2004–2019 can be divided into three main growth periods, namely 2004–2009, 2010–2012, and 2013–2019, the first growth period being from 2004 to 2009, the number of publications totaled 6,749, accounting for 23.97% of all publications; from 2010 to 2012, the number of publications totaled 8,236, accounting for 17.56% of all publications; and from 2013 to 2019, the number of publications totaled 22,473, accounting for 58.47% of all publications. Of these, 2006 was the first year of sharp growth with an annual growth rate of 19.97%, 2009 was the second year of sharp growth with an annual growth rate of 17.64%, and 2008 was the third year of sharp growth with an annual growth rate of 16.09%. In the last 5 years, 2019 has also shown a sharp growth trend with a growth rate of 14.34%. Notably, in 2010 and 2013, there was negative growth with the growth rate of −3.39 and −1.45%. In the last 10 years, depression research has become one of the most valuable areas of human research. It can also be noted that the number of publications in the field of depression in these 10 countries has been increasing year after year.

Second, the analysis is conducted from the perspective of national development, divided into developed and developing countries, as shown in the orange bar chart in Figure 2, where the horizontal coordinate is year and the vertical coordinate is total publications, comparing the article productivity variability between developed and developing countries. The top 10 most productive countries in the field of depression globally include nine developed countries and one developing country, respectively. During the period 2004–2019, 34,631 papers were published in developed countries and 3,802 papers were published in developing countries, with developed countries accounting for 90.11% of the 38,433 articles and developing countries accounting for 9.89%, and the total number of publications in developed countries was about 9 times higher than that in developing countries. During the period 2004–2019, the number of publications in developed countries showed negative growth in 2 years (2010 and 2013) with growth rates of −3.39 and −1.45%, respectively. The rest of the years showed positive growth with growth rates of 1.52% (2005), 19.97 (2006), 8.11 (2007), 12.70 (2008), 17.64 (2009), 13.22 (2011), 10.17 (2012), 16.09 (2014), 10.46 (2015), 4.10 (2016), 1.59 (2017), 3.91 (2018), and 14.34 (2019), showing three periods of positive growth: 2004–2009, 2011–2012, and 2014–2019, with the highest growth rate of 19.97% in 2006. Recent years have also shown a higher growth trend, with a growth rate of 14.34% in 2019. It is worth noting that developing countries have been showing positive growth in the number of articles in the period 2004–2019, with annual growth rates of 81.25 (2005), 17.24 (2006), 35.29 (2007), 19.57 (2008), 65.45 (2009), 13.19 (2010), 29.13 (2011), 54.89 (2012), 12.14 (2013), 36.36 (2014), 14.92 (2015), 16.02 (2016), 10.24 (2017), 21.17 (2018), and 31.37 (2019), with the highest growth rate of 81.25% in 2005. In the field of depression research, developed countries are still the main force and occupy an important position.

Further, 10 countries with the highest productivity in the field of depression are compared, total publications in the vertical coordinate, and the colored scatter plot contains 10 colored dots, representing 10 different countries. On the one hand, the variability of the contributions of different countries in the same time frame can be compared horizontally. On the other hand, it is possible to compare vertically the variability of the growth of different countries over time. Among them, USA, with about 40.29% of the world's publications in the field of depression, has always been a leader in the field of depression with its rich research results. Peoples R China, as the only developing country, ranks 3rd in the top 10 countries with high production of research papers in the field of depression, and Peoples R China's research in the field of depression has shown a rapid growth trend, and by 2016, it has jumped to become the 2nd largest country in the world, with the number of published papers increasing year by year, which has a broad prospect and great potential for development.

### Distribution of Periodicals

Table 2 lists the top 15 journals in order of number of journal co-citations. In the field of depression, the top 15 cited journals accounted for 19.06% of the total number of co-citations, nearly one in five of the total number of journal co-citations. In particular, the top 3 journals were ARCH GEN PSYCHIAT (ARCHIVES OF GENERAL PSYCHIATRY), J AFFECT DISORDERS (JOURNAL OF AFFECTIVE DISORDERS), and AM J PSYCHIAT (AMERICAN JOURNAL OF PSYCHIATRY), with co-citation counts of 20,499, 20,302, and 20,143, with co-citation rates of 2.09, 2.07, and 2.06%, respectively. The main research area of ARCH GEN PSYCHIAT is Psychiatry; the main research area of the journal J AFFECT DISORDERS is Neurosciences and Neurology, Psychiatry; AM J PSYCHIAT is the main research area of Psychiatry, and the three journals have “psychiatry” in common, making them the most frequently co-cited journals in the field of depression.

**Table 2:** The top 15 co-cited journals.

**Rank**	**Journal**	**TP**	**TP R (%)**	**BC**
1	ARCH GEN PSYCHIAT	20,499	2.09	0.02
2	J AFFECT DISORDERS	20,302	2.07	0.07
3	AM J PSYCHIAT	20,143	2.06	0.01
4	BIOL PSYCHIAT	15,538	1.59	0.04
5	BRIT J PSYCHIAT	15,109	1.54	0.01
6	PSYCHOL MED	13,183	1.35	0
7	J CLIN PSYCHIAT	12,778	1.30	0.01
8	JAMA-J AM MED ASSOC	11,868	1.21	0.02
9	ACTA PSYCHIAT SCAND	10,171	1.04	0
10	LANCET	9,179	0.94	0
11	PSYCHIAT RES	8,231	0.84	0
12	PLOS ONE	7,704	0.79	0
13	NEUROPSYCHOPHARMACOL	7,616	0.78	0.01
14	DIAGN STAT MAN MENT	7,553	0.77	0
15	PSYCHOSOM MED	6,920	0.71	0.01

*TP, total publications; TP R (%), the ratio of the amount of the journal's publications to the total publications; BC, betweenness centrality*.

**Figure 3:**
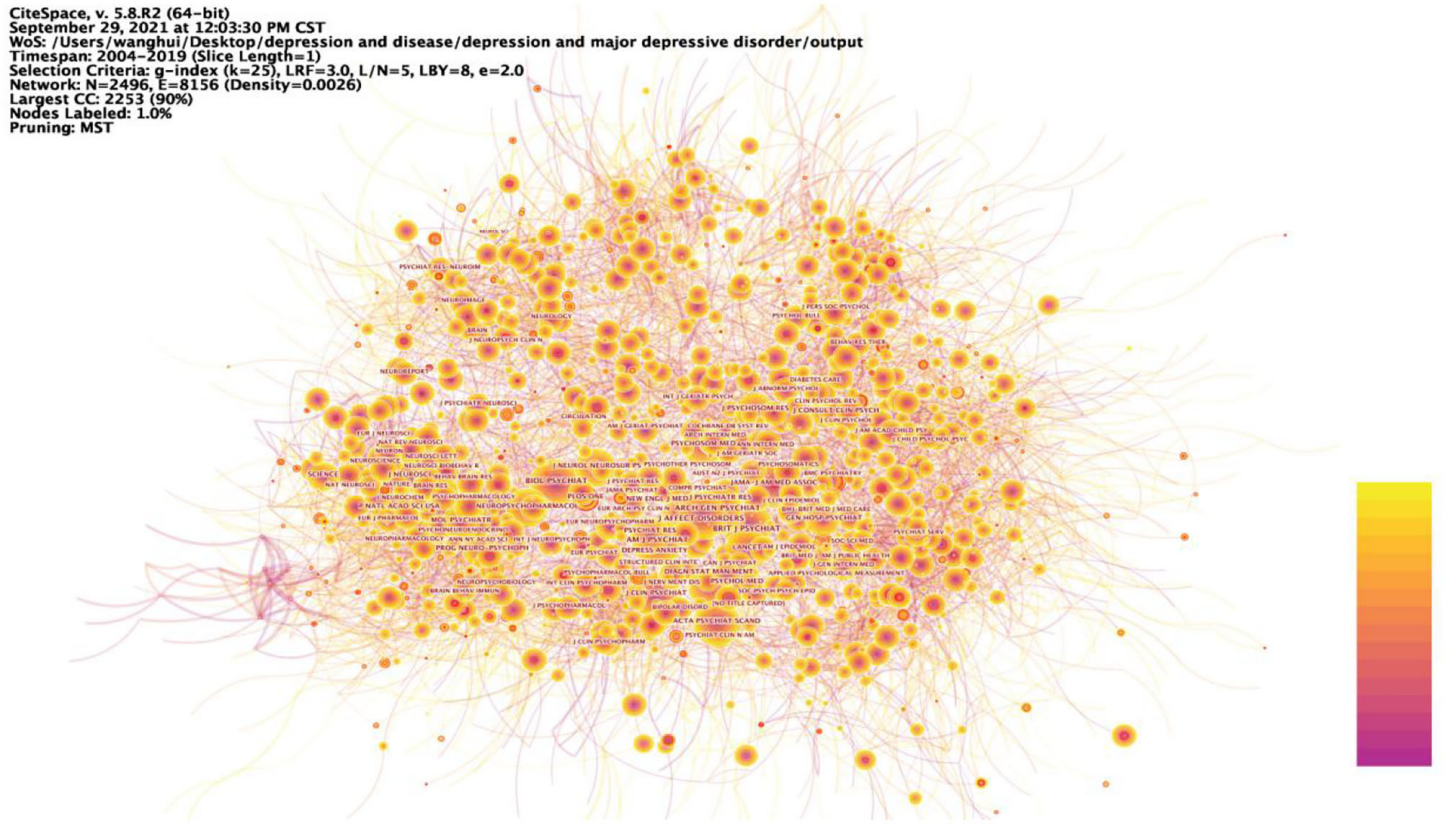
Prominent journals involved in depression. The betweenness centrality of a node in the network measures the importance of the position of the node in the network. Two types of nodes may have high betweenness centrality scores: (1) Nodes that are highly connected to other nodes, (2) Nodes are positioned between different groups of nodes. The lines represent the link between two different nodes.

Figure 3 shows the network relationship graph of the cited journals from 2004 to 2019. The figure takes g-index as the selection criteria, the scale factor *k* = 25 to include more nodes. Each node of the graph represents each journal, the node size represents the number of citation frequencies, the label size represents the size of the betweenness centrality of the journal in the network, and the links between journals represent the co-citation relationships. The journal co-citation map reflects the structure of the journals, indicating that there are links between journals and that the journals include similar research topics. These journals included research topics related to neuroscience, psychiatry, neurology, and psychology. The journal with betweenness centrality size in the top 1 was ARCH GEN PSYCHIAT, with betweenness centrality size of 0.07, and impact shadows of 14.48. ARCH GEN PSYCHIAT, has research themes of Psychiatry. In all, these journals in Figure 3 occupy an important position in the journal's co-citation network and have strong links with other journals.

### Distribution of Categories

Table 3 lists the 15 most popular categories in the field of depression research during the period 2004–2019. In general, the main disciplines involved are neuroscience, psychology, pharmacy, medicine, and health care, which are closely related to human life and health issues. Of these, psychiatry accounted for 20.78%, or about one-five, making it the most researched category. The study of depression focuses on neuroscience, reflecting the essential characteristics of depression as a category of mental illness and better reflecting the fact that depression is an important link in the human public health care. In addition, Table 3 shows that the category with the highest betweenness centrality is Neuroscience, followed by Public, Environment & Occupational Health, and then Pharmacology & Pharmacy, with betweenness centrality of 0.16, 0.13, and 0.11, respectively. It is found that the research categories of depression are also centered on disciplines such as neuroscience, public health and pharmacology, indicating that research on depression requires a high degree of integration of multidisciplinary knowledge and integration of information from various disciplines in order to have a more comprehensive and in-depth understanding of the depression.

**Table 3:** The top 15 productive categories, 2004–2019.

**Rank**	**WoS categories**	**TP**	**TP R (%)**	**BC**
1	PSYCHIATRY	19,804	20.78	0.07
2	NEUROSCIENCES & NEUROLOGY	12,355	12.96	0.01
3	CLINICAL NEUROLOGY	7,297	7.66	0.03
4	NEUROSCIENCES	6,848	7.19	0.16
5	PSYCHOLOGY	4,284	4.49	0.09
6	PHARMACOLOGY & PHARMACY	3,124	3.28	0.11
7	GENERAL &INTERNAL MEDICINE	2,682	2.81	0
8	MEDICINE, GENERAL, & INTERNAL	2,532	2.66	0.06
9	PSYCHOLOGY, CLINICAL	2,340	2.46	0
10	PUBLIC, ENVIRONMENTAL, & OCCUPATIONAL HEALTH	2,087	2.19	0.13
11	GERIATRICS & GERONTOLOGY	2,046	2.15	0.01
12	GERONTOLOGY	1,558	1.63	0
13	NURSING	1,454	1.53	0.07
14	HEALTH CARE SCIENCES & SERVICES	1,380	1.45	0.08
15	SCIENCE & TECHNOLOGY—OTHER TOPICS	1,301	1.37	0.04

*TP, total publications; TP R (%), the ratio of the amount of the category's publications to the total publications; BC, betweenness centrality*.

**Figure 4:**
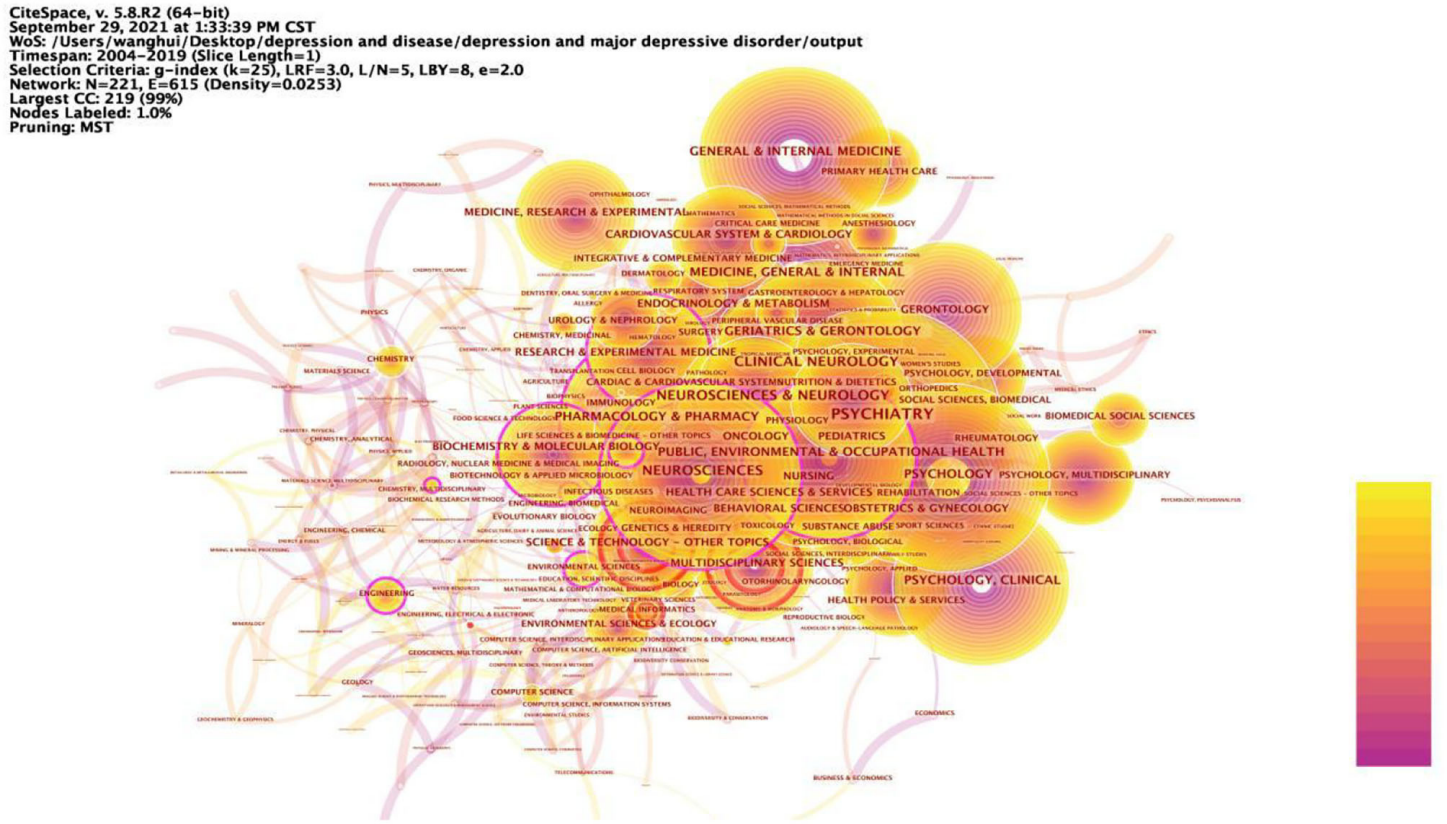
Prominent categories involved in depression, 2004–2019. The betweenness centrality of a node in the network measures the importance of the position of the node in the network. Two types of nodes may have high betweenness centrality scores: (1) Nodes that are highly connected to other nodes, (2) Nodes are positioned between different groups of nodes. The lines represent the link between two different nodes.

Figure 4 shows the nine categories with the betweenness centrality in the category research network, with Neuroscience being the node with the highest betweenness centrality in this network, meaning that Neuroscience is most strongly linked to all research categories in the field of depression research. Depression is a debilitating psychiatric disorder with mood disorders. It is worth noting that the development of depression not only has psychological effects on humans, but also triggers many somatic symptoms that have a bad impact on their daily work and life, giving rise to the second major mediating central point of research with public health as its theme. The somatization symptoms of depression often manifest as abnormalities in the cardiovascular system, and many studies have looked at the pathology of the cardiovascular system in the hope of finding factors that influence the onset of depression, mechanisms that trigger it or new ways to treat it. Thus, depression involves not only the nervous system, but also interacts with the human cardiovascular system, for example, and the complexity of depression dictates that the study of depression is an in-depth study based on complex systems.

### Author Statistics

The results of the analysis showed that there were many researchers working in the field of depression over the past 16 years, and 63 of the authors published at least 30 articles related to depression. Table 4 lists the 15 authors with the highest number of articles published. It includes the rank of the number of articles published, author, country, number of articles published in depression-related studies, total number of articles included in Web of Science, total number of citations, average number of citations, and H-index. According to the statistics, seven of the top 15 authors are from USA, three from the Netherlands, one from Canada, one from Australia, one from New Zealand, one from Italy, and one from Germany. From this, it can be seen that these productive authors are from developed countries, thus it can be inferred that developed countries have a better research environment, more advanced research technology and more abundant research funding. The evaluation indicators in the author co-occurrence network are frequency, betweenness centrality and time of first appearance. The higher the frequency, i.e., the higher the number of collaborative publications, the more collaboration, the higher the information dissemination rate, the three authors with the highest frequency in this author co-occurrence network are MAURIZIO FAVA, BRENDA W. J. H. PENNINX, MADHUKAR H. TRIVEDI; the higher the betweenness centrality, i.e., the closer the relationship with other authors, the more collaboration, the higher the information dissemination rate, the three authors with the highest betweenness centrality are the three authors with the highest betweenness centrality are MICHAEL E. THASE, A. JOHN RUSH; the time of first appearance, i.e., the longer the influence generated by the author's research, the higher the information dissemination rate; in addition, the impact factor and citations can also reflect the information dissemination efficiency of the authors.

**Table 4:** The top 15 authors in network of co-authorship, 2004–2019.

**Rank**	**Author**	**Country**	**Year**	**BC**	**TP**	**AP**	**DP (%)**	**TC**	**CPP (%)**	**H-index**
1	MAURIZIO FAVA	USA	2006	0.09	250	1,073	23.30	51,094	47.62	105
2	BRENDA W. J. H PENNINX	Netherlands	2008	0.05	184	725	25.38	70,413	97.12	129
3	MADHUKAR H. TRIVEDI	USA	2006	0.02	151	802	18.83	40,171	50.09	93
4	MICHAEL E. THASE	USA	2006	0.21	141	980	14.39	54,423	55.53	109
5	PIM CUIJPERS	Netherlands	2006	0.1	113	618	18.28	41,429	67.04	108
6	CHARLES F.	USA	2007	0.05	100	531	18.83	13,890	26.16	63
7	A. JOHN RUSH	USA	2006	0.11	94	913	10.30	64,237	70.36	116
8	MICHAEL BERK	Australia	2007	0.04	94	677	13.88	27,532	40.67	79
9	DAVID C. STEFFENS	USA	2006	0.03	86	471	18.26	19,156	40.67	72
10	BERNHARD T. BAUNE	New Zealand	2008	0.04	82	554	14.80	33,365	60.23	76
11	ALESSANDRO SERRETTI	Italy	2007	0.02	75	858	8.74	21,563	25.13	69
12	AARTJAN T. F. BEEKMAN	Netherlands	2007	0.03	74	700	10.57	32,972	47.10	92
13	VOLKER AROLT	Germany	2006	0.01	73	622	11.74	20,165	32.42	77
14	ROGER S. MCINTYRE	Canada	2014	0.09	73	770	9.48	21,639	28.10	72
15	DAVID MISCHOULON	USA	2008	0.02	71	300	23.67	7,104	23.68	44

*BC, betweenness centrality; TP, total publications; AP, publications in all areas; DP (%), the ratio of the publications about depression in 2004–2019 to the publications about all areas in all times; TC, total citation; CPP (%), citations per publication*.

**Figure 5:**
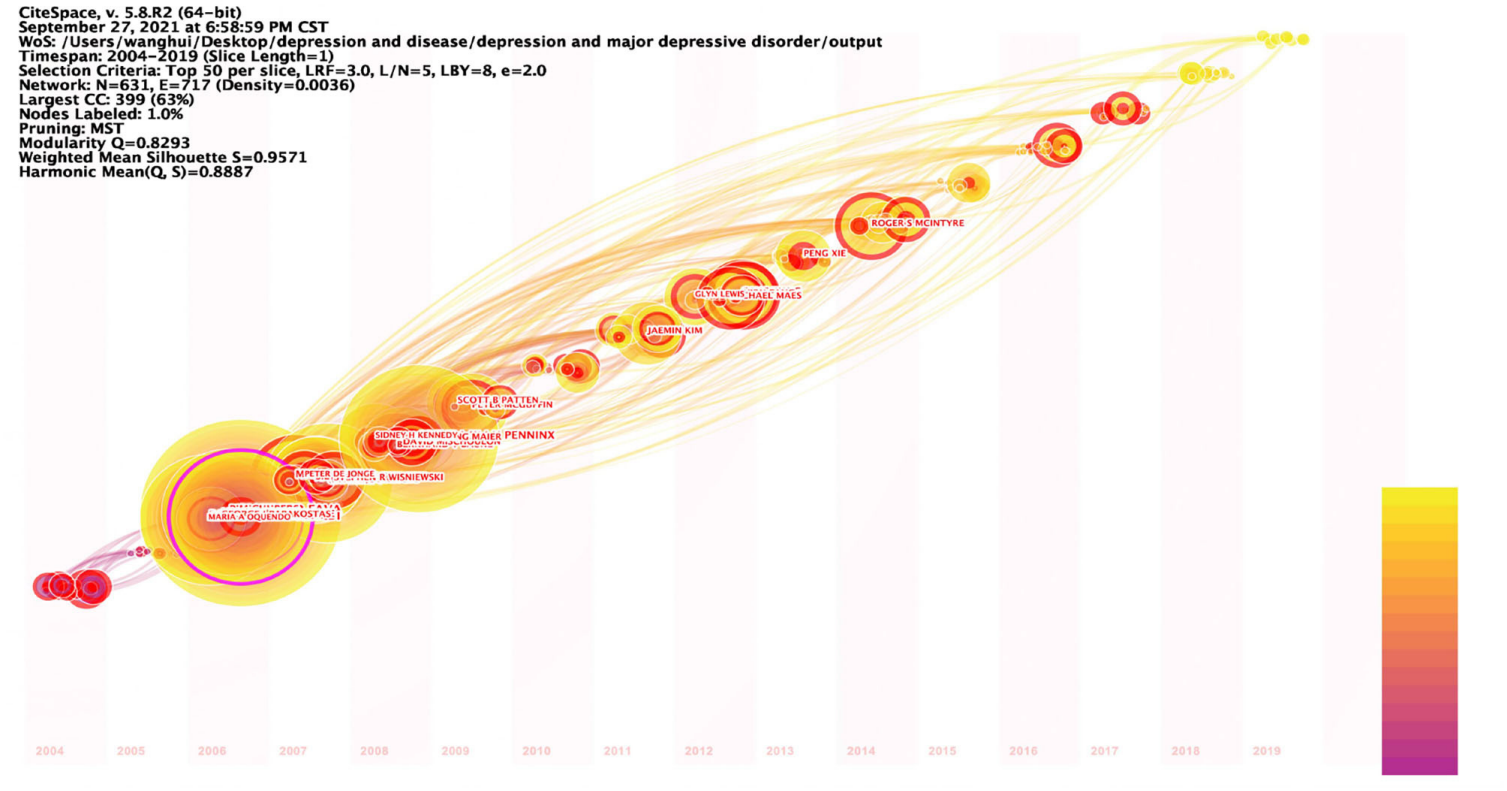
Timezone view of the author's co-existing network in depression, 2004–2019. The circle represents the author, the time zone in which the circle appears is the year in which the author first published in this study dataset, the radius of the circle represents the frequency of appearance, the color represents the different posting times, the lines represent the connections between authors, and the time zone diagram shows the evolution of author collaboration.

The timezone view (Figure 5) in the author co-occurrence network clearly shows the updates and interactions of author collaborations, for example. All nodes are positioned in a two-dimensional coordinate with the horizontal axis of time, and according to the time of first posting, the nodes are set in different time zones, and their positions are sequentially upward with the time axis, showing a left-to-right, bottom-up knowledge evolution diagram. The time period 2004–2019 is divided into 16 time zones, one for each year, and each circle in the figure represents an author, and the time zone in which the circle appears is the year when the author first published an article in the data set of this study. The closer the color, the warmer the color, the closer the time, the colder the color, the older the era, the thickness of an annual circle, and the number of articles within the corresponding time division is proportional, the dominant color can reflect the relative concentration of the emergence time, the nodes appear in the annual circle of the red annual circle, that is, on behalf of the hot spot, the frequency of being cited was or is still increasing sharply. Nodes with purple outer circles are nodes with high betweenness centrality. The time zone view demonstrates the growth of author collaboration in the field, and it can be found from the graph that the number of author collaborations increases over time, and the frequency of publications in the author collaboration network is high; observe that the thickness of the warm annual rings in the graph is much greater than the thickness of the cold annual rings, which represents the increase of collaboration in time; there are many authors in all time zones, which indicates that there are many research collaborations and achievements in the field, and the field is in a period of collaborative prosperity. The linkage relationship between the sub-time-periods can be seen by the linkage relationship between the time periods, and it can be found from the figure that there are many linkages in the field in all time periods, which indicates that the author collaboration in the field of depression research is strong.

### Institutional Statistics

**Figure 6:**
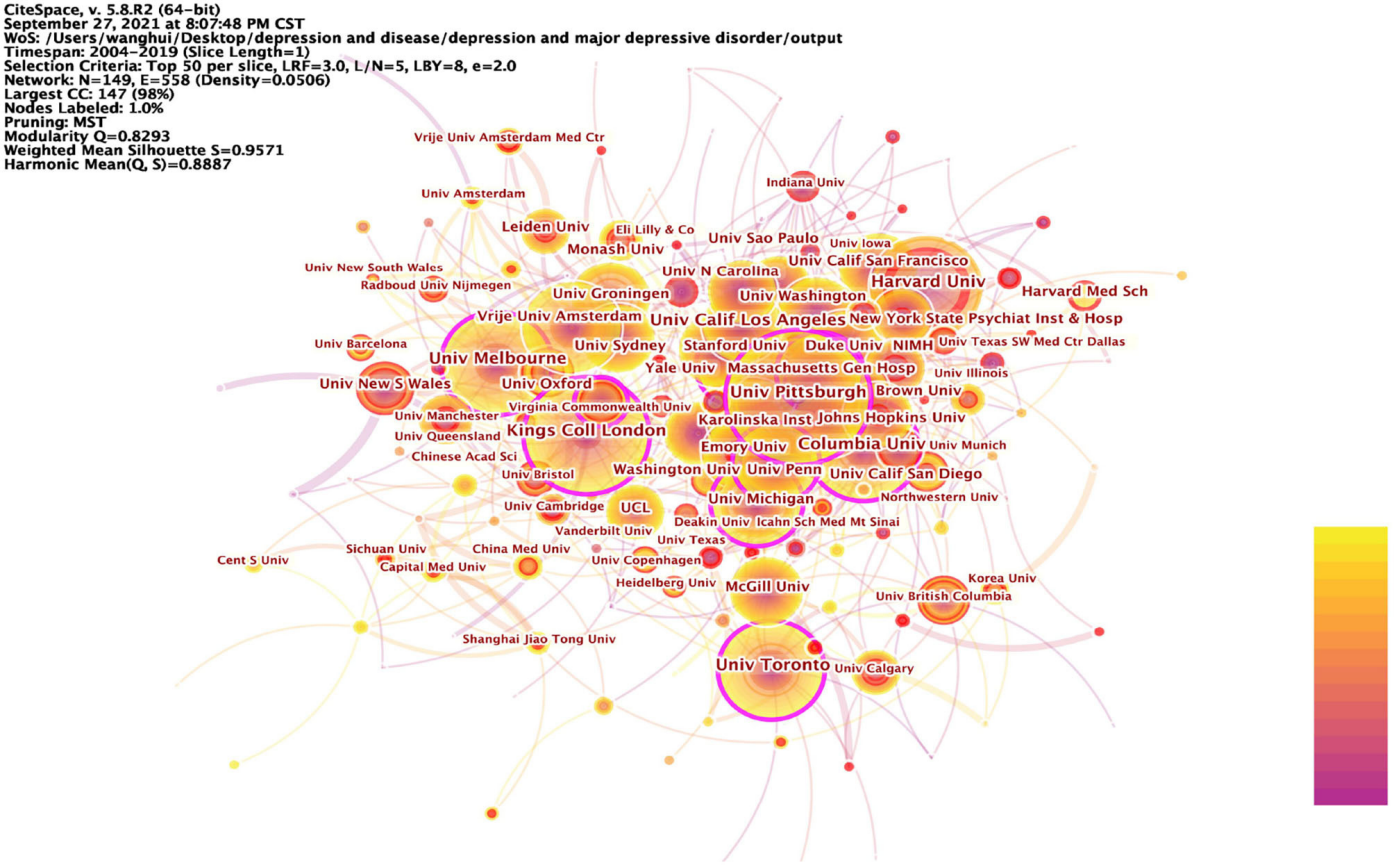
Prominent institutions involved in depression, 2004–2019. The betweenness centrality of a node in the network measures the importance of the position of the node in the network. Two types of nodes may have high betweenness centrality scores: (1) Nodes that are highly connected to other nodes, (2) Nodes are positioned between different groups of nodes. The lines represent the link between two different nodes.

Table 5 lists the top 15 research institutions in network of co-authors' institutions. These include 10 American research institutions, two Netherlands research institutions, one UK research institution, one Canadian research institution and one Australian research institution, all of which, according to the statistics, are from developed countries. Of these influential research institutions, 66.7% are from USA. Figure 6 shows the collaborative network with these influential research institutions as nodes. Kings Coll London (0.2), Univ Michigan (0.17), Univ Toronto (0.15), Stanford Univ (0.14), Univ Penn (0.14), Univ Pittsburgh (0.14), Univ Melbourne (0.12), Virginia Commonwealth Univ (0.12), Columbia Univ (0.1), Duke Univ (0.1), Massachusetts Gen Hosp (0.1), Vrije Univ Amsterdam (0.1), with betweenness centrality >0.1. Kings Coll London has a central place in this collaborative network and is influential in the field of depression research. Table 6 lists the 15 institutions with the strong burst strength. The top 3 institutions are all from USA. Univ Copenhagen, Univ Illinois, Harvard Med Sch, Boston Univ, Univ Adelaide, Heidelberg Univ, Univ New South Wales, and Icahn Sch Med Mt Sinai have had strong burst strength in recent years. It suggests that these institutions may have made a greater contribution to the field of depression over the course of this year and more attention could be paid to their research.

**Table 5:** The top 15 institutions in network of co-authors' institutions, 2004–2019.

**Rank**	**Institutions**	**Country**	**TP**	**BC**
1	Univ Pittsburgh	USA	1,008	0.14
2	Kings Coll London	UK	908	0.2
3	Harvard Univ	USA	907	0.01
4	Univ Toronto	Canada	813	0.15
5	Columbia Univ	USA	800	0.1
6	Univ Melbourne	Australia	678	0.12
7	Univ Calif Los Angeles	USA	671	0.05
8	Univ Penn	USA	623	0.14
9	Vrije Univ Amsterdam	Netherlands	613	0.1
10	Duke Univ	USA	612	0.1
11	Univ Washington	USA	608	0.03
12	Univ Michigan	USA	608	0.17
13	Massachusetts Gen Hosp	USA	599	0.1
14	Univ Groningen	Netherlands	557	0.07
15	Stanford Univ	USA	557	0.14

*TP, total publications; BC, betweenness centrality*.

**Table 6:** The top 15 institutions with the strongest citation bursts, 2004–2019.

**Institutions**	**Year**	**Strength**	**Begin**	**End**	**2004–2019**
Univ Texas	2004	90.32	2004	2007	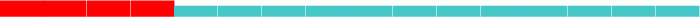
Indiana Univ	2004	43.03	2004	2010	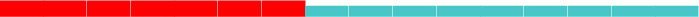
Eli Lilly & Co.	2004	41.93	2004	2010	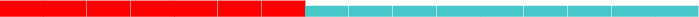
Univ Munich	2004	40.34	2004	2012	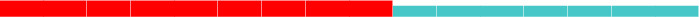
Cornell Univ	2004	34.45	2004	2008	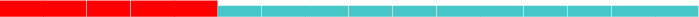
Univ Texas SW Med Ctr Dallas	2004	62.3	2008	2013	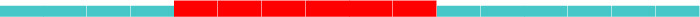
Charite	2004	36.61	2010	2014	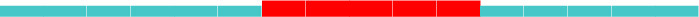
Univ Copenhagen	2004	36.72	2014	2019	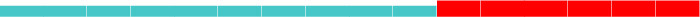
Univ Illinois	2004	36.13	2015	2019	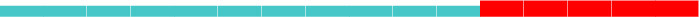
Harvard Med Sch	2004	122.08	2016	2019	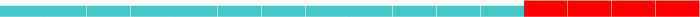
Boston Univ	2004	37.25	2016	2019	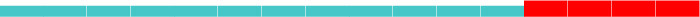
Univ Adelaide	2004	35.86	2016	2019	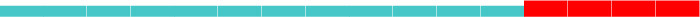
Heidelberg Univ	2004	33.1	2016	2019	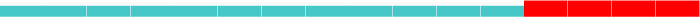
Univ New South Wales	2004	47.6	2017	2019	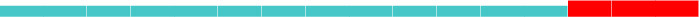
Icahn Sch Med Mt Sinai	2004	43.05	2017	2019	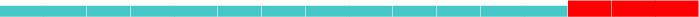

*Burst denote the citation burst strength; blue thin lines denote the whole period of 2004962019, which provide a useful means to trace the development of research focus; the location and length of red thick lines denote the start and end time during the whole period of the bursts and how long the burst lasts*.

Summing up the above analysis, it can be seen that the research institutions in USA are at the center of the depression research field, are at the top of the world in terms of quantity and quality of research, and are showing continuous growth in vitality. Research institutions in USA, as pioneers among all research institutions, lead and drive the development of depression research and play an important role in cutting-edge research in the field of depression.

### Article Citations

Table 7 lists the 16 articles that have been cited more than 1,000 times within the statistical range of this paper from 2004 to 2019. As can be seen from the table, the most cited article was written by Dowlati et al. from Canada and published in BIOLOGICAL PSYCHIATRY 2010, which was cited 2,556 times. In addition, 11 of these 16 highly cited articles were from the USA. Notably, two articles by Kroenke, K as first author appear in this list, ranked 7th and 11th, respectively. In addition, there are three articles from Canada, one article from Switzerland, and one article from the UK. And interestingly, all of these countries are developed countries. It can be reflected that developed countries have ample research experience and high quality of research in the field of depression research. On the other hand, it also reflects that depression is a key concern in developed countries. These highly cited articles provide useful information to many researchers and are of high academic and exploratory value.

**Table 7:** The top 15 frequency cited articles, 2004–2019.

**Rank**	**Title**	**Author**	**Year**	**Country**	**TP**	**Journal**
1	A meta-analysis of cytokines in major depression (22)	Dowlati, Y	2010	Canada	2,556	BIOLOGICAL PSYCHIATRY
2	Evaluation of outcomes with citalopram for depression using measurement-based care in STAR*D: implications for clinical practice (23)	Trivedi, MH	2006	USA	2,354	AMERICAN JOURNAL OF PSYCHIATRY
3	Deep brain stimulation for treatment-resistant depression (24)	Mayberg, HS	2005	Canada	2,314	NEURON
4	Depression, chronic diseases, and decrements in health: results from the World Health Surveys (3)	Moussavi, S	2006	Switzerland	2,219	LANCET
5	A randomized trial of an N-methyl-D-aspartate antagonist in treatment-resistant major depression (25)	Zarate, CA	2007	USA	2,088	ARCHIVES OF GENERAL PSYCHIATRY
6	The molecular neurobiology of depression (26)	Krishnan, V	2008	USA	1,691	NATURE
7	The PHQ-8 as a measure of current depression in the general population (27)	Kroenke, K	2009	USA	1,602	JOURNAL OF AFFECTIVE DISORDERS
8	5-HTTLPR polymorphism impacts human cingulate-amygdala interactions: a genetic susceptibility mechanism for depression (28)	Pezawas, L	2005	USA	1,447	NATURE NEUROSCIENCE
9	Resting-state functional connectivity in major depression: Abnormally increased contributions from subgenual cingulate cortex and thalamus (29)	Greicius, MD	2007	USA	1,403	BIOLOGICAL PSYCHIATRY
10	Sustained hippocampal chromatin regulation in a mouse model of depression and antidepressant action (30)	Tsankova, NM	2006	USA	1,242	NATURE NEUROSCIENCE
11	An Ultra-Brief Screening Scale for anxiety and depression: the PHQ-4 (31)	Kroenke, K	2009	USA	1,173	PSYCHOSOMATICS
12	Fluoxetine, cognitive-behavioral therapy, and their combination for adolescents with depression—Treatment for adolescents with depression study (TADS) randomized controlled trial (32)	March, J	2004	USA	1,155	JAMA-JOURNAL OF THE AMERICAN MEDICAL ASSOCIATION
13	Epidemiology of major depressive disorder—Results from the National Epidemiologic Survey on Alcoholism and Related Conditions (33)	Hasin, DS	2005	USA	1,155	ARCHIVES OF GENERAL PSYCHIATRY
14	Cognition and depression: current status and future directions (34)	Gotlib, IH	2010	USA	1,131	ANNUAL REVIEW OF CLINICAL PSYCHOLOGY, VOL. 6
15	Antenatal risk factors for postpartum depression: a synthesis of recent literature (35)	Robertson, E	2004	Canada	1,084	GENERAL HOSPITAL PSYCHIATRY
16	Prevalence of depression, anxiety, and adjustment disorder in oncological, hematological, and palliative-care settings: a meta-analysis of 94 interview-based studies (36)	Mitchell, AJ	2011	UK	1,072	LANCET ONCOLOGY

*TP, total publications (citations)*.

## Research Hotspots Ang Frontiers

### Keyword Analysis

**Figure 7:**
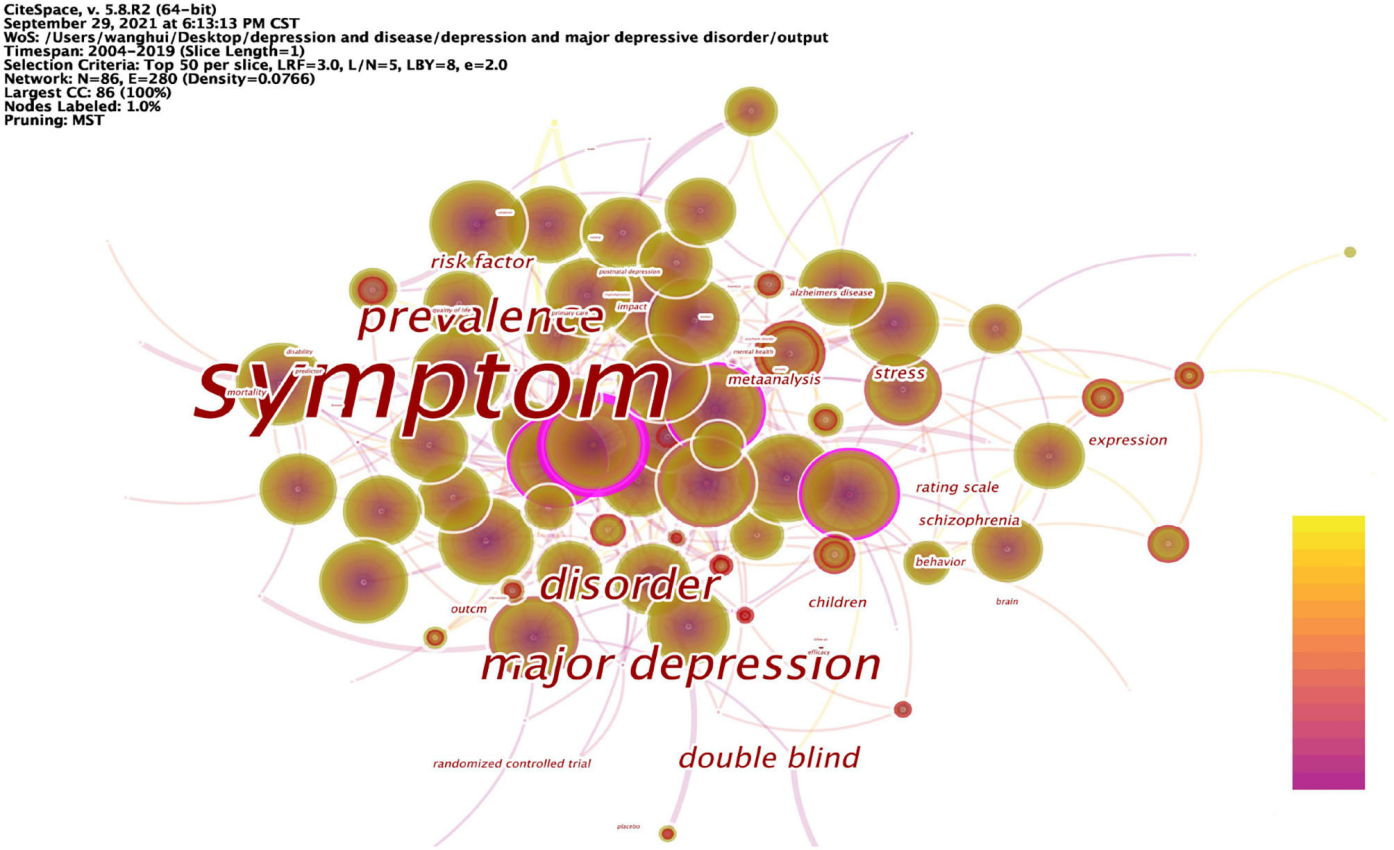
Keyword co-occurrence network in depression, 2004–2019.

The keyword analysis of depression yielded the 25 most frequent keywords in Table 8 and the keyword co-occurrence network in Figure 7. Also, the data from this study were detected by burst, the 25 keywords with the strongest burst strength were obtained in Table 9. These results bring out the popular and cutting-edge research directions in the field clearly.

**Table 8:** Top 25 frequent keywords in the period of 2004–2019.

**Rank**	**Keywords**	**Year**	**Count**	**BC**
1	Symptom	2004	7,335	0.6
2	Disorder	2004	7,071	0.25
3	Major depression	2004	5,883	0.28
4	Prevalence	2004	5,455	0.27
5	Meta-analysis	2004	3,212	0.08
6	Anxiety	2004	3,153	0.02
7	Risk	2004	3,040	0.01
8	Scale	2004	2,779	0.03
9	Association	2004	2,759	0
10	Quality of life	2004	2,756	0.04
11	Health	2004	2,753	0
12	Risk factor	2004	2,439	0.12
13	Stress	2004	2,056	0.11
14	Validity	2004	1,873	0.03
15	Validation	2004	1,819	0.02
16	Mental health	2004	1,817	0.04
17	Women	2004	1,802	0.03
18	Double blind	2004	1,760	0.18
19	Brain	2004	1,626	0.07
20	Population	2004	1,605	0.01
21	Disease	2004	1,500	0.02
22	Impact	2004	1,499	0.06
23	Primary care	2004	1,477	0.04
24	Mood	2004	1,459	0.01
25	Efficacy	2004	1,456	0.04

*Count, number of times the article has been cited; BC, betweenness centrality*.

**Table 9:** Top 25 keywords with strongest citation bursts in the period of 2004–2019.

**Keywords**	**Year**	**Strength**	**Begin**	**End**	**2004–2019**
Fluoxetine	2004	111.2	2004	2007	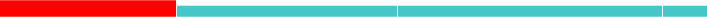
Community	2004	110.08	2004	2007	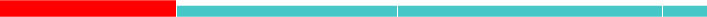
Antidepressant treatment	2004	94.28	2009	2011	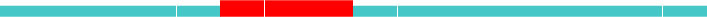
Severity	2004	88.35	2014	2019	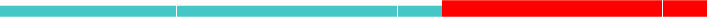
Meta-analysis	2004	86.42	2017	2019	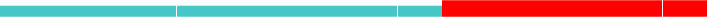
People	2004	85.33	2015	2017	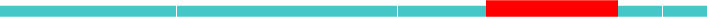
Follow up	2004	84.46	2004	2013	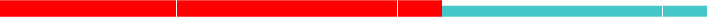
Expression	2004	79.78	2017	2019	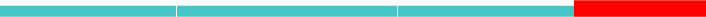
Trial	2004	72.79	2006	2008	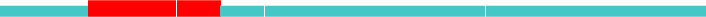
Epidemiology	2004	66.93	2012	2015	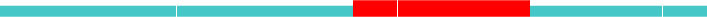
Model	2004	64.4	2013	2019	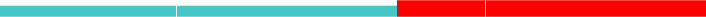
United States	2004	63.4	2010	2012	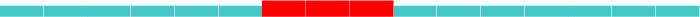
Adolescent	2004	63.13	2014	2015	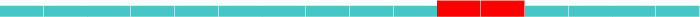
Serotonin reuptake inhibitor	2004	62.21	2008	2009	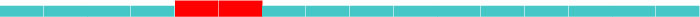
Late life depression	2004	59.71	2009	2010	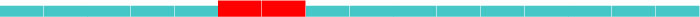
Disability	2004	52.29	2007	2008	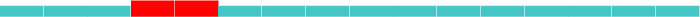
Myocardial infarction	2004	50.59	2008	2009	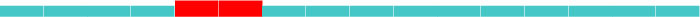
Placebo	2004	49.32	2006	2007	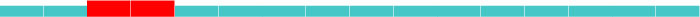
Hospital anxiety	2004	43.33	2008	2013	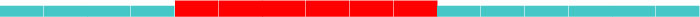
Illness	2004	42.3	2004	2005	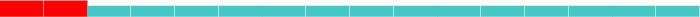
Major depression	2004	42.22	2012	2013	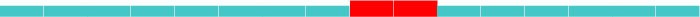
Dementia	2004	41.81	2005	2007	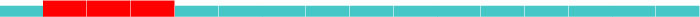
Prefrontal cortex	2004	40.93	2016	2019	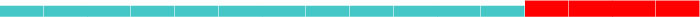
Psychiatric disorder	2004	35.34	2004	2008	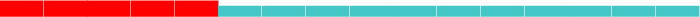
Management	2004	35.08	2016	2017	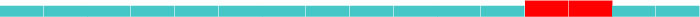

*Burst denote the citation burst strength; blue thin lines denote the whole period of 2004–2019, which provide a useful means to trace the development of research focus; the location and length of red thick lines denote the start and end time during the whole period of the bursts and how long the burst lasts*.

The articles on depression during 2004–2019 were analyzed in 1-year time slices, and the top 25 keywords with the highest frequency of occurrence were selected from each slice to obtain the keyword network shown in Table 8. The top 25 keywords with the highest frequencies were: symptom, disorder, major depression, prevalence, meta-analysis, anxiety, risk, scale, association, quality of life, health, risk factor, stress, validity, validation, mental health, women, double blind, brain, population, disease, impact, primary care, mood, and efficacy. High-frequency nodes respond to popular keywords and are an important basis for the field of depression research.

Figure 7 shows the co-occurrence network mapping of keywords regarding depression research. Each circle in the figure is a node representing a keyword, and the greater the betweenness centrality, the more critical the position of the node in the network. The top 10 keywords in terms of betweenness centrality are: symptom (0.6), major depression (0.28), prevalence (0.27), disorder (0.25), double blind (0.18), risk factor (0.12), stress (0.11), children (0.1), schizophrenia (0.1), and expression (0.1). Nodes with high betweenness centrality reflect that the keyword forms a co-occurrence relationship with multiple other keywords in the domain. A higher betweenness centrality indicates that it is more related to other keywords, and therefore, the node plays an important role in the study. Relatively speaking, these nodes represent the main research directions in the field of depression; they are also the key research directions in this period, and to a certain extent, represent the research hotspots in this period.

Burst detection was performed on the keywords, and the 25 keywords with the strongest strength were extracted, as shown in Table 9. These keywords contain: fluoxetine, community, follow up, illness, psychiatric disorder, dementia, trial, placebo, disability, serotonin reuptake inhibitor, myocardial infarction, hospital anxiety, antidepressant treatment, late life depression, United States, epidemiology, major depression, model, severity, adolescent, people, prefrontal cortex, management, meta-analysis, and expression. The keywords that burst earlier include fluoxetine (2004), community (2004), follow up (2004), illness (2004), and psychiatric disorder (2004), are keywords that imply that researchers focused on themes early in the field of depression. As researchers continue to explore, the study of depression is changing day by day, and the keywords that have burst in recent years are people (2015), prefrontal cortex (2016), management (2016), meta-analysis (2017), and expression (2017). Reflecting the fact that depression research in recent years has mainly focused on human subjects, the focus has been on the characterization of populations with depression onset. The relationship between depression and the brain has aroused the curiosity of researchers, what exactly are the causes that trigger depression and what are the effects of depression for the manifestation of depression have caused a wide range of discussions in the research community, and the topics related to it have become the most popular studies and have been the focus of research in recent years. All of these research areas showed considerable growth, indicating that research into this area is gaining traction, suggesting that it is becoming a future research priority. The keywords with the strongest burst strength are fluoxetine (111.2), community (110.08), antidepressant treatment (94.28), severity (88.35), meta-analysis (86.42), people (85.33), and follow up (84.46). The rapid growth of research based on these keywords indicates that these topics are the most promising and interesting. The keywords that has been around the longest burst are follow up (2004–2013), model (2013–2019), hospital anxiety (2008–2013), severity (2014–2019), and psychiatric disorder (2004–2008), researchers have invested a lot of research time in these research directions, making many research results, and responding to the exploratory value and significance of research on these topics. At the same time, the longer duration of burst also proves that these research directions have research potential and important value.

### Research Hotspots

Hotspots must mainly have the characteristics of high frequency, high betweenness centrality, strong burst, and time of emergence can be used as secondary evaluation indicators. The higher the number of occurrences, the higher the degree of popularity and attention. The higher betweenness centrality means the greater the influence and the higher the importance. Nodes with strong burst usually represent key shift nodes and need to be focused on. The time can be dynamically adjusted according to the target time horizon of the analysis. Thus, based on the results of statistical analysis, it is clear that the research hotspots in the field of depression can be divided into four main areas: etiology (external factors, internal factors), impact (quality of life, disease symptoms, co-morbid symptoms), treatment (interventions, drug development, care modalities), and assessment (population, size, symptoms, duration of disease, morbidity, mortality, effectiveness).

### Etiology

Risk factors for depression include a family history of depression, early life abuse and neglect, and female sexuality and recent life stressors. Physical illnesses also increase the risk of depression, particularly increasing the prevalence associated with metabolic (e.g., cardiovascular disease) and autoimmune disorders.

Research on the etiology of depression can be divided into internal and external factors. In recent years, researchers have increasingly focused on the impact of external factors on depression. Depression is influenced by environmental factors related to social issues, such as childhood experiences, social interactions, and lifestyles. Adverse childhood experiences are risk factors for depression and anxiety in adolescence (37) and are a common pathway to depression in adults (38). Poor interpersonal relationships with classmates, family, teachers, and friends increase the prevalence of depression in adolescents (39). Related studies assessed three important, specific indicators of the self-esteem domain: social confidence, academic ability, and appearance (40). The results suggest that these three dimensions of self-esteem are key risk factors for increased depressive symptoms in Chinese adolescents. The vulnerability model (41) suggests that low self-esteem is a causal risk factor for depression, and low self-esteem is thought to be one of the main causes of the onset and progression of depression, with individuals who exhibit low self-esteem being more likely to develop social anxiety and social withdrawal, and thus having a sense of isolation (42), which in turn leads to subsequent depression. Loneliness predicts depression in adolescents. Individuals with high levels of loneliness experience more stress and tension from psychological and physical sources in their daily lives, which, combined with insufficient care from society, can lead to depression (43). A mechanism of association exists between life events and mood disorders, with negative life events being directly associated with depressive symptoms (44). In a cross-sectional study conducted in Shanghai, the prevalence of depression was higher among people who worked longer hours, and daily lifestyle greatly influenced the prevalence of depression (45). A number of studies in recent years have presented a number of interesting ideas, and they suggest that depression is related to different environmental factors, such as temperature, sunlight hours, and air pollution. Environmental factors have been associated with suicidal behavior. Traffic noise is a variable that triggers depression and is associated with personality disorders such as depression (46). The harmful effects of air pollution on mental health, inhalation of air pollutants can trigger neuroinflammation and oxidative stress and induce dopaminergic neurotoxicity. A study showed that depression was associated with an increase in ambient fine particulate matter (PM2.5) (47).

Increased inflammation is a feature of many diseases and even systemic disorders, such as some autoimmune diseases [e.g., type 1 diabetes (48) or rheumatoid arthritis (49)] and infectious diseases [e.g., hepatitis and sepsis (50)], are associated with an inflammatory response and have been found to increase the risk of depression. A growing body of evidence supports a bidirectional association between depression and inflammatory processes, with stressors and pathogens leading to excessive or prolonged inflammatory responses when combined with predisposing factors (e.g., childhood adversity and modifying factors such as obesity). The resulting illnesses (e.g., pain, sleep disorders), depressive symptoms, and negative health (e.g., poor diet, sedentary lifestyle) may act as mediating pathways leading to inflammation and depression. In terms of mechanistic pathways, cytokines induce depression by affecting different mood-related processes. Elevated inflammatory signals can dysregulate the metabolism of neurotransmitters, damaging neurons, and thus altering neural activity in the brain. In addition cytokines can modulate depression by regulating hormone levels. Inflammation can have different effects on different populations depending on individual physiology, and even lower levels of inflammation may have a depressive effect on vulnerable individuals. This may be due to lower parasympathetic activity, poorer sensitivity to glucocorticoid inhibitory feedback, a greater response to social threat in the anterior oral cortex or amygdala and a smaller hippocampus. Indeed, these are all factors associated with major depression that can affect the sensitivity to the inhibitory consequences of inflammatory stimuli.

### Impact

Depression triggers many somatization symptoms, which can manifest as insomnia, menopausal syndrome, cardiovascular problems, pain, and other somatic symptoms. There is a link between sleep deprivation and depression, with insomnia being a trigger and maintenance of depression, and more severe insomnia and chronic symptoms predicting more severe depression. Major depression is considered to be an independent risk factor for the development of coronary heart disease and a predictor of cardiovascular events (51). Patients with depression are extremely sensitive to pain and have increased pain perception (52) and is associated with an increased risk of suicide (53, 54), and generally the symptoms of these pains are not relieved by medication.

Studies have shown that depression triggers an inflammatory response, promoting an increase in cytokines in response to stressors vs. pathogens. For example, mild depressive symptoms have been associated with an amplified and prolonged inflammatory response (55, 56) following influenza vaccination in older adults and pregnant women. Among women who have recently given birth, those with a lifetime history of major depression have greater increases in both serum IL-6 and soluble IL-6 receptors after delivery than women without a history of depression (57). Pro-inflammatory agents, such as interferon-alpha (IFN-alpha), for specific somatization disorders [e.g., hepatitis C or malignant melanoma (58, 59)], although effective for somatic disorders, pro-inflammatory therapy often leads to psychiatric side effects. Up to 80% of patients treated with IFN-α have been reported to suffer from mild to moderate depressive symptoms.

### Treatment

Clinical trials have shown better antidepressant treatment with anti-inflammatory drugs compared to placebo, either as monotherapy (60, 61) or as an add-on treatment (62–65) to antidepressants (66, 67). However, findings like whether NSAIDs can be safely used in combination with antidepressants are controversial. Patients with depression often suffer from somatic co-morbidities, which must be included in the benefit/risk assessment. It is important to consider the type of medication, duration of treatment, and dose, and always balance the potential treatment effect with the risk of adverse events in individual patients. Depression, childhood adversity, stressors, and diet all affect the gut microbiota and promote gut permeability, another pathway that enhances the inflammatory response, and effective depression treatment may have profound effects on mood, inflammation, and health. Early in life gut flora colonization is associated with hypothalamic-pituitary-adrenal (HPA) axis activation and affects the enteric nervous system, which is associated with the risk of major depression, gut flora dysbiosis leads to the onset of TLR4-mediated inflammatory responses, and pro-inflammatory factors are closely associated with depression. Clinical studies have shown that in the gut flora of depressed patients, pro-inflammatory bacteria such as Enterobacteriaceae and Desulfovibrio are enriched, while short-chain fatty acid producing bacteria are reduced, and some of these bacterial taxa may transmit peripheral inflammation into the brain via the brain-gut axis (68). In addition, gut flora can affect the immune system by modulating neurotransmitters (5-hydroxytryptamine, gamma-aminobutyric acid, norepinephrine, etc.), which in turn can influence the development of depression (69). Therefore, antidepressant drugs targeting gut flora are a future research direction, and diet can have a significant impact on mood by regulating gut flora.

As the molecular basis of clinical depression remains unclear, and treatments and therapeutic effects are limited and associated with side effects, researchers have worked to discover new treatment modalities for depression. High-amplitude low-frequency musical impulse stimulation as an additional treatment modality seems to produce beneficial effects (70). Studies have found electroconvulsive therapy to be one of the most effective antidepressant treatment therapies (71). Physical exercise can promote molecular changes that lead to a shift from a chronic pro-inflammatory to an anti-inflammatory state in the peripheral and central nervous system (72). Aromatherapy is widely used in the treatment of central nervous system disorders (73). By activating the parasympathetic nervous system, qigong can be effective in reducing depression (74). The exploration of these new treatment modalities provides more reference options for the treatment of depression.

### Assessment

Large-scale assessments of depression have found that the probability of developing depression varies across populations. Depression affects some specific populations more significantly, for example: adolescents, mothers, and older adults. Depression is one of the disorders that predispose to adolescence, and depression is associated with an increased risk of suicide among college students (75). Many women develop depression after childbirth. Depression that develops after childbirth is one of the most common complications for women in the postpartum period (76). The health of children born to mothers who suffer from postpartum depression can also be adversely affected (77). Depression can cause many symptoms within the central nervous system, especially in the elderly population (78).

Furthermore, one of the most consistent findings of the association between inflammation and depression is the elevated levels of peripheral pro-inflammatory markers in depressed individuals, and peripheral pro-inflammatory marker levels can also be used as a basis for the assessment of depressed patients. Studies have shown that the following pro-inflammatory markers have been found to be at increased levels in depressed individuals: CRP (79, 80), IL-6 (22, 79, 81, 82), TNF–α, and interleukin-1 receptor antagonist (IL-1ra) (79, 82), however, this association is not unidirectional and the subsequent development of depression also increases pro-inflammatory markers (82, 83). These biomarkers are of great interest, and depressed patients with increased inflammatory markers may represent a relatively drug-resistant population.

### Frontier Analysis

The exploration and analysis of frontier areas of depression were based on the results of the analysis of the previous section on keywords. According to the evaluation index and analysis idea of this study, the frontier research topics need to have the following four characteristics: low to medium frequency, strong burst, high betweenness centrality, and the research direction in recent years. Therefore, combining the results of keyword analysis and these characteristics, it can be found that the frontier research on depression also becomes clear.

### Research on Depression Characterized by Psychosexual Disorders

Exploration of biological mechanisms based on depression-associated neurological disorders and analysis of depression from a neurological perspective have always been the focus of research. Activation of neuroinflammatory pathways may contribute to the development of depression (84). A research model based on the microbial-gut-brain axis facilitates the neurobiology of depression (85). Some probiotics positively affect the central nervous system due to modulation of neuroinflammation and thus may be able to modulate depression (86). The combination of environmental issues and the neurobiological study of depression opens new research directions (46).

### Research on Relevant Models of Depression

How to develop a model that meets the purpose of the study determines the outcome of the study and has become the direction that researchers have been exploring in recent years. Martínez et al. (87) developed a predictive model to assess factors that modify the treatment pathway for postpartum depression. Nie et al. (88) extended the work on predictive modeling of treatment-resistant depression to establish a predictive model for treatment-resistant depression. Rational modeling methods and behavioral testing facilitate a more comprehensive exploration of depression, with richer studies and more scientifically valid findings.

### Research and Characterization of the Depressed Patient Population

Current research on special groups and depression has received much attention. In a study of a group of children, 4% were found to suffer from depression (89). The diagnosis and treatment of mental health disorders is an important component of pediatric care. Second, some studies of populations with distinct characteristics have been based primarily on female populations. Maternal perinatal depression is also a common mental disorder with a prevalence of over 10% (90). In addition, geriatric depression is a chronic and specific disorder (91). Studies based on these populations highlight the characteristics of the disorder more directly than large-scale population explorations and are useful for conducting extended explorations from specific to generalized.

### Somatic Comorbidities Associated With Depression

Depression often accompanies the onset and development of many other disorders, making the study of physical comorbidities associated with depression a new landing place for depression research. Depression is a complication of many neurological or psychopathological disorders. Depression is a common co-morbidity of glioblastoma multiforme (92). Depression is an important disorder associated with stroke (93). Chronic liver disease is associated with depression (94). The link between depressive and anxiety states and cancer has been well-documented (95). In conclusion, depression is associated with an increased risk of lung, oral, prostate, and skin cancers, an increased risk of cancer-specific death from lung, bladder, breast, colorectal, hematopoietic system, kidney, and prostate cancers, and an increased risk of all-cause mortality in lung cancer patients. The early detection and effective intervention of depression and its complications has public health and clinical implications.

### Research on Mechanisms of Depression

Research based on the mechanisms of depression includes the study of disease pathogenesis, the study of drug action mechanisms, and the study of disease treatment mechanisms. Research on the pathogenesis of depression has focused more on the study of the hypothalamic-pituitary-adrenal axis. Social pressure can change the hypothalamic-pituitary-adrenal axis (96). Studies on the mechanism of action of drugs are mostly based on their effects on the central nervous system. The antidepressant effects of Tanshinone IIA are mediated by the ERK-CREB-BDNF pathway in the hippocampus of mice (97). Research on the mechanisms of depression treatment has also centered on the central nervous system. It has been shown that the vagus nerve can transmit signals to the brain that can lead to a reduction in depressive behavior (98).

## Conclusion

In this study, based on the 2004–2019 time period, this wealth of data is effectively integrated through data analysis and processing to reproduce the research process in a particular field and to co-present global trends in homogenous fields while organizing past research.

Journals that have made outstanding contributions in this field include ARCH GEN PSYCHIAT, J AFFECT DISORDERS and AM J PSYCHIAT. PSYCHIATRY, NEUROSCIENCES & NEUROLOGY and CLINICAL NEUROLOGY are the three most popular categories. The three researchers with the highest number of articles were MAURIZIO FAVA (USA), BRENDA W. J. H. PENNINX (NETHERLANDS) and MADHUKAR H TRIVEDI (USA). Univ Pittsburgh (USA), Kings Coll London (UK) and Harvard Univ (USA) are three of the most productive and influential research institutions. A Meta-Analysis of Cytokines in Major Depression, Evaluation of outcomes with citalopram for depression using measurement-based care in STAR*D: Implications for clinical practice and Deep brain stimulation for treatment-resistant depression are key articles. Through keyword analysis, a distribution network centered on depression was formed. Although there are good trends in the research on depression, there are still many directions to be explored in depth. Some recommendations regarding depression are as follows.

(1) The prevention of depression can be considered by focusing on treating external factors and guiding the individual.

Faced with the rising incidence of depression worldwide and the difficulty of treating depression, researchers can think more about how to prevent the occurrence of depression. Depressed moods are often the result of stress, not only social pressures on the individual, but also environmental pressures in the developmental process, which in turn have an unhealthy relationship with the body and increase the likelihood of depression. The correlation between external factors and depression is less well-studied, but the control of external factors may be more effective in the short term than in the long term, and may be guided by self-adjustment to avoid major depressive disorder.

(2) The measurement and evaluation of the degree of depression should be developed in the direction of precision.

In the course of research, it has been found that the Depression Rating Scale is mostly used for the detection and evaluation of depression. This kind of assessment is more objective, but it still lacks accuracy, and the research on measurement techniques and methods is less, which is still at a low stage. Patients with depression usually have a variety of causes, conditions, and duration of illness that determine the degree of depression. Therefore, whether these scales can truly accurately measure depression in depressed patients needs further consideration. Accurate measurement is an important basis for evidence-based treatment of depression, and thus how to achieve accurate measurement of depression is a research direction that researchers can move toward.

Therefore, there is an urgent need for further research to address these issues.

A systematic analysis of research in the field of depression in this study concludes that the distribution of countries, journals, categories, authors, institutions, and citations may help researchers and research institutions to establish closer collaboration, develop appropriate publication plans, grasp research hotspots, identify valuable research ideas, understand current emerging research, and determine research directions. In addition, there are still some limitations that can be overcome in future work. First, due to the lack of author and address information in older published articles, it may not be possible to accurately calculate their collaboration; second, although the data scope of this paper is limited to the Web of Science, it can adequately meet our objectives.

## Data Availability Statement

The original contributions presented in the study are included in the article/supplementary material, further inquiries can be directed to the corresponding author/s.

## Author Contributions

HW conceived and designed the analysis, collected the data, performed the analysis, and wrote the paper. XT, XW, and YW conceived and designed the analysis. All authors contributed to the article and approved the submitted version.

## Funding

This work was supported by the National Natural Science Foundation of China under Grant No. 81973495.

## Conflict of Interest

The authors declare that the research was conducted in the absence of any commercial or financial relationships that could be construed as a potential conflict of interest.

## Publisher's Note

All claims expressed in this article are solely those of the authors and do not necessarily represent those of their affiliated organizations, or those of the publisher, the editors and the reviewers. Any product that may be evaluated in this article, or claim that may be made by its manufacturer, is not guaranteed or endorsed by the publisher.
